# Novel Medial Application of a Locking Attachment Washer in Managing Hoffa Fracture Non-union With Peri-Implant Femoral Fracture: A Case Report

**DOI:** 10.7759/cureus.82664

**Published:** 2025-04-20

**Authors:** Eng Kee Tan, Mohd Afiq Muhamed Fuad, Ashraf Hakim Ab Halim

**Affiliations:** 1 Orthopedics, Universiti Putra Malaysia, Serdang, MYS

**Keywords:** femoral condyle fractures, femoral fractures, intramedullary fracture fixation, non-union, periprosthetic fractures

## Abstract

Non-union of Hoffa fractures is an uncommon but challenging complication due to the intra-articular nature and limited bone surface area available for fixation. When compounded by a peri-implant distal femur fracture, treatment becomes significantly more complex, particularly in the presence of previous hardware.

We present a rare case of a 59-year-old male with a chronic non-union medial Hoffa fracture complicated by a peri-implant fracture at the proximal end of a distal femoral locking plate. Definitive management involved implant removal, retrograde intramedullary femoral nailing using the RFN-Advanced™ system, bone grafting via the Reamer-Irrigator-Aspirator technique, and the novel medial application of a lateral locking attachment washer (LAW) to stabilize the Hoffa fragment. This configuration provided dual-plane stability and facilitated successful fracture union.

Radiological union was observed at three months postoperatively, with the patient achieving a 0-130 degrees range of motion and a Knee Society Score of 90/100 at one year. No complications were reported, and functional recovery was excellent.

The medial deployment of a lateral LAW in conjunction with a retrograde femoral nail represents a biomechanically sound and surgically viable solution for complex distal femoral fracture patterns involving Hoffa non-union and peri-implant fractures. This technique broadens the applicability of the LAW and may serve as a reference for future cases with similar dual pathology.

## Introduction

Hoffa fractures are coronal plane fractures of the distal femoral condyle, most commonly resulting from high-energy mechanisms such as motor vehicle accidents (80.5%) and falls (9.1%), accounting for 8.7% to 13% of distal femur fractures [1]. Conservative management of Hoffa fractures is typically associated with poor outcomes, while internal fixation has demonstrated improved results. However, non-union of Hoffa fractures remains a rare but serious complication that can impair knee function significantly. They are an uncommon but challenging complication due to their intra-articular nature and limited bone surface area available for fixation. Articular defects need to be addressed diligently while ensuring adequate compression and stability to achieve union.

Peri-implant distal femur fractures are known complications associated with previous fixation, often resulting from stress concentration at the plate end, especially in osteoporotic bone or after repeat trauma. In this report, we describe a rare and complex case involving a chronic non-union of a medial Hoffa fracture (Letenneur type III), initially treated with a lateral distal femoral locking plate, complicated by a subsequent peri-implant fracture. We highlight the novel use of a lateral locking attachment washer applied medially in combination with retrograde femoral nailing as a successful strategy for achieving fracture union and functional recovery.

## Case presentation

A 59-year-old male presented to the emergency department following a road traffic accident with pain and deformity in the right distal thigh. He had undergone surgery six years earlier for a medial Hoffa fracture and tibial plateau fracture using a distal femoral locking plate. No additional distal femur injuries were noted at that time. His pre-injury range of motion (ROM) was limited to 0-60 degrees, and he was unable to engage in prolonged physical activity.

**Figure 1:**
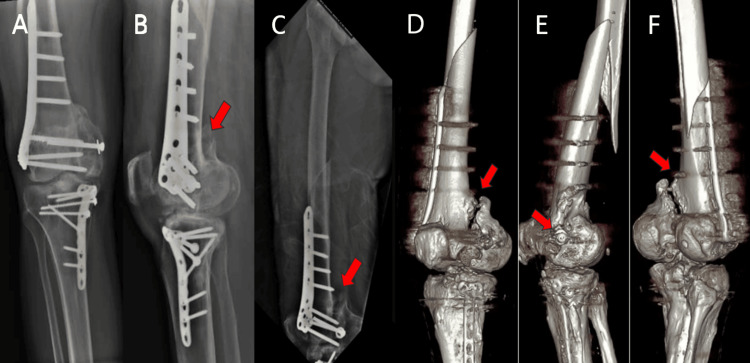
Preoperative plain radiograph and three-dimensional computed tomography (A) Plain radiograph of right knee (AP view); (B) Plain radiograph of right knee (lateral view); (C) Plain radiograph of right femur (lateral view); (D) 3D CT of right knee (posterior aspect); (E) 3D CT of right knee (medial aspect); (F) 3D CT of right knee (posterolateral aspect) The red arrow indicates the presence of a gap over the previous medial Hoffa fragment.

On examination, he was hemodynamically stable with no other associated injuries. Radiographs showed a peri-implant fracture at the proximal end of the distal femur plate and a clear non-union at the medial Hoffa fracture site. A 3D CT scan confirmed the chronic non-union and detailed the fracture morphology (Figure 1).

The patient was initially stabilized with skeletal traction. After informed consent, surgical intervention was planned, involving implant removal, retrograde femoral nailing using the RFN-Advanced™ system, bone grafting, and medial placement of a lateral LAW. Under combined spinal-epidural anesthesia, a sub-vastus approach provided exposure for implant removal and non-union site preparation. The medial Hoffa fragment was mobile despite previous fixation. All fibrous tissue was debrided, and the site was prepared for bone grafting.

**Figure 2:**
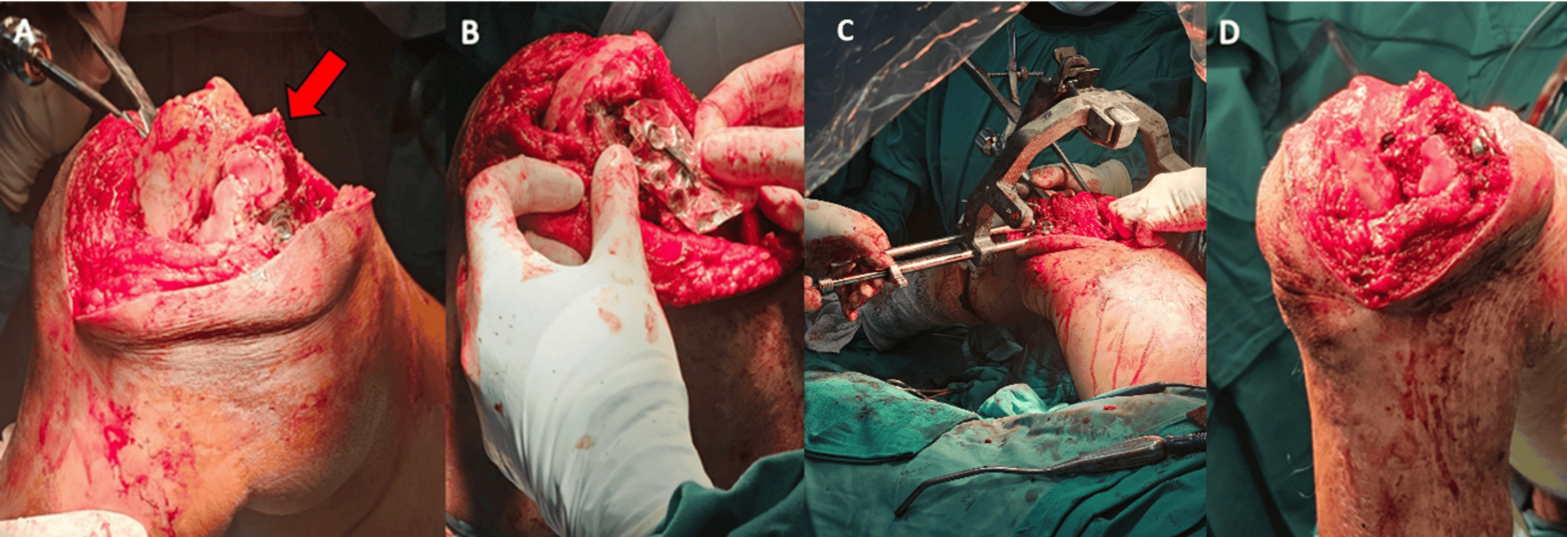
Intraoperative images and findings (A) Exposed femoral condyle of right knee indicating significant fracture gap at the nonunion site (red arrow); (B) Sampling of ipsilateral LAW within sterile wrap; (C) Insertion of the RFN-Advance^TM^ and placement of the LAW on the medial side; (D) Final placement of LAW on the medial epicondyle of right femur LAW: locking attachment washer

A 30 cc intramedullary bone graft was harvested using a Reamer Irrigator-Aspirator (RIA) and placed into the non-union site. The fragment was reduced and temporarily stabilized with K-wires and a compression screw. Notably, degenerative changes and chondral injuries were observed within the joint. Intraoperatively, exposure of the joint cartilage was kept to a minimum duration, and manipulation of the fracture fragments was performed with instruments placed judiciously on non-weight-bearing areas of the bone. Retrograde nailing was performed, and a lateral LAW was applied medially using the RFN-Advanced™ jig system, which accommodates 180-degree guided screw placement (Figure 2). Two variable-angle locking screws were inserted medially to further secure the Hoffa fragment. The construct provided adequate stability on fluoroscopic assessment and intraoperative manipulation.

**Figure 3:**
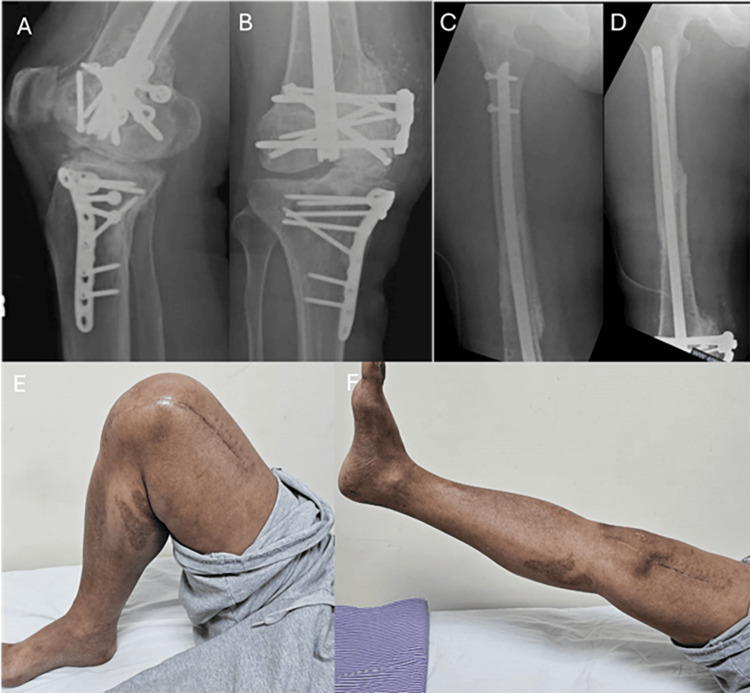
Follow-up radiographs and knee range of motion after four months (A) Plain radiograph of right knee (lateral view) indicating union of Hoffa fracture; (B) Plain radiograph of right knee (AP view) indicating union of Hoffa fracture; (C) Plain radiograph of right femur (lateral view) indicating union of femur fracture; (D) Plain radiograph of right femur (AP view) indicating union of femur fracture; (E) and (F) Clinical photograph demonstrating full right knee range of motion

Postoperatively, the surgical wound healed uneventfully. Radiographs confirmed progressive union at both the peri-implant and Hoffa fracture sites, with full union achieved by three months. At one year postoperatively, the patient had regained independent ambulation and improved ROM from 0 to 130 degrees (Figure 3). His Knee Society Score was 90/100 at both six-month and one-year follow-ups.

## Discussion

Hoffa fractures pose significant surgical challenges due to their intra-articular location and the difficulty in achieving stable fixation. Non-union further complicates the scenario and is rarely reported in the literature. This case was further complicated by a peri-implant fracture, requiring a comprehensive and innovative fixation strategy.

Retrograde intramedullary nailing with a medial application of a lateral LAW allowed simultaneous stabilization of both the diaphyseal peri-implant fracture and the intra-articular Hoffa fragment. This construct enabled relative stability across the femoral shaft while providing interfragmentary compression for the non-union site. The use of a long distal femoral locking plate might have compromised screw placement for the Hoffa fragment and increased the risk of future peri-implant fractures.

The literature offers limited guidance on the treatment of non-union Hoffa fractures. Nandy et al. described successful outcomes using lag screws and a neutralization plate, while others used dynamic compression plates or L-shaped plates with bone grafting [2,3]. To our knowledge, this is the first report describing the use of a retrograde femoral nail in combination with a medially applied LAW to manage such a complex dual pathology.

The RFN-Advanced™ system with LAW adjunct provides higher biomechanical strength, including a 42% increase in load to failure and a 30.5% increase in axial stiffness [4]. Although initially designed for lateral application, our case demonstrates that medial use is feasible and effective, expanding its potential applications. Compared to locking plates, retrograde nails are associated with lower infection rates and higher union rates in distal femur fractures [5].

The LAW is a novel fixation option that was designed to improve implant anchorage in patients undergoing retrograde femoral nail fixation with distal fractures. Its design allows for two variable-angle interlocking screws to be placed through the LAW and the two distal screw holes of the RFN-Advanced™ nail [4]. The LAW is designed to be placed against the lateral femoral epicondyle, and its usage on the medial epicondyle, such as in this case, has not yet been reported in literature. In this case report, the authors’ novel utilization of the LAW on the medial side required the accommodation of an ipsilateral design LAW that was aligned to the guidance jig and oriented to purchase the medial Hoffa with its screws. Biomechanically, the presence of variable-angle screws allowed for a large variation of screw placements with minimal soft tissue and bony manipulation. The guidance jig also allowed for screws to interlock between the LAW and RFN-Advanced™ nail. Its ability to integrate with the RFN-Advanced™ nail and its locking screw design allowed for increased construct stability. Care has to be taken during the placement of the LAW, and a plate designed for contralateral limb usage could be considered to ensure appropriate placement with minimal soft tissue irritation and prominent implants. In this case, though an ipsilateral LAW was selected, the outcome was good with no implant prominence or soft tissue complications postoperatively.

## Conclusions

This case report presents a rare and complex orthopedic challenge involving a chronic medial Hoffa fracture non-union compounded by a peri-implant distal femur fracture. The innovative use of a retrograde femoral nail in conjunction with a medially applied LAW - a technique not previously documented in the literature - demonstrated successful fracture union and excellent functional recovery. This approach underscores the importance of adaptable fixation strategies in complex orthopedic trauma and may offer a valuable reference for future management of similar cases where conventional methods may fall short.
